# Secondary findings in hereditary cancer genes after germline genetic testing – systematic review of literature

**DOI:** 10.1007/s00439-025-02746-w

**Published:** 2025-04-28

**Authors:** Eva Avsec, Ana Blatnik, Mateja Krajc

**Affiliations:** 1 Department of Clinical Cancer Genetics, Institute of Oncology Ljubljana, Zaloška 2, Ljubljana, 1000 Slovenia; 2 Faculty of Medicine, University of Ljubljana, Korytkova 2, Ljubljana, 1000 Slovenia

## Abstract

In the last decade the increasing use of germline genetic testing has led to frequent discoveries of secondary findings (SF) in hereditary cancer (HC) genes. Disclosure and clinical management of such findings are still not clearly defined and raise many ethical, clinical, and practical questions. This systematic review is focused on frequency of reported SF in HC genes across different populations as well as summarizing current guidelines, recommendations, and actual clinical practice about reporting and managing SF in HC genes. A systematic literature search according to the PRISMA guidelines was performed on the electronic database PubMed from inception to June 2024. 30 research papers involving almost 150,000 patients were reviewed. The reported frequencies of SF in HC genes varied between 0.4 and 3.1%. The majority of patients agreed to receive SF for medically actionable genes. Management and surveillance of patients after disclosure of SF in HC genes were rarely reported, but the limited data show no regret of receiving such results as well as diagnoses of early-stage cancer in patients participating in recommended surveillance programs related to SF. A substantial number of carriers of highly penetrant pathogenic variants in HC genes is discovered by reporting SF after germline genetic testing with next-generation sequencing. Additional information about the impact of SF disclosure on individuals and health care systems is needed to optimize the integration of SF into clinical care.

## Introduction

Over the last few years, the use of panel, exome, and genome analyses through next-generation sequencing (NGS) for clinical purposes has resulted in increased detection of SF. There are other terms for SF used in the literature such as - incidental finding, additional finding or unsolicited finding. According to the American College of Medical Genetics and Genomics (ACMG) (Miller et al. 2021) the term incidental finding refers to results of a genetic test that is unexpected, unrelated to the initial indication for genetic testing and could not have been anticipated before the genetic test. In contrast, the term SF refers to a result of deliberate screening for pathogenic/likely pathogenic variants in genes that are a part of a defined list, but are not related to the primary indication for the genetic test.

Different authors of larger population studies from all over the world (Amendola et al. 2015; Retterer et al. 2016; Hart et al. 2019; Gordon et al. 2020; Van Rooij et al. 2020; Haverfield et al. 2021; Horiuchi et al. 2021; Jensson et al. 2023; Nolan et al. 2024a) describe the frequency of all SF to be between 1 and 7% after whole exome or genome sequencing, but a lot remains unknown in regard of frequency, relevance to health condition and actionability of SF in HC genes.

In the last decade, the extensive use of exome sequencing, genome sequencing or multi-gene panel analyses has uncovered variations in cancer predisposition genes not directly related to the initial focus of genetic testing. Additionally, somatic tumour panels can reveal germline SF in HC genes (Raymond et al. 2016; Pujol et al. 2019). This consequently results in increased detection of potentially clinically significant germline pathogenic/likely pathogenic variants beyond the expected yield of targeted germline testing. Management of SF in HC genes regarding primary and secondary prevention for patients and their families is still widely debated and presents a clinical as well as an ethical and legal challenge for clinicians.

## Summary of current recommendations for disclosure of SF

In 2013, ACMG (Green et al. 2013) recommended reporting likely pathogenic and pathogenic variants in genes linked to conditions that are medically actionable before symptoms appear, proposing a list of 56 genes. In 2021, they updated their recommendations (Miller et al. 2021) for reporting SF in whole exome sequencing (WES) and whole genome sequencing (WGS). They emphasized that selected genes should be medically actionable, have a highly penetrant and clear disease-associated phenotype. They clarified that the ACMG SF gene list is a minimum list intended to reduce morbidity and mortality, not an inclusive list of all potentially actionable genetic results. They also acknowledged that some laboratories might include additional genes in their reports.

The most recent update to the ACMG SF list was published in April 2023 (Miller et al. 2023) and includes 81 genes, with 28 genes related to cancer phenotypes. The list of recommended genes is now set to be updated annually, with the ongoing goal of maintaining it as a minimum list.

At present, there are only a few equivalent guidelines in Europe for managing of SF in HC genes. These include the recommendations from the European Society of Human Genetics (Van El et al. 2013) and recommendations from French Society of Predictive and Personalized Medicine (SFMPP) (Pujol et al. 2018), but the recommendations from ACMG are still widely used in Europe.

SFMPP’s working group published guidelines for reporting SF cancer genes in 2018. Their proposed minimum list of genes that needs to be reported unless patients opt out includes 36 HC genes. French recommendations emphasize the importance of patient autonomy - the need for the patient to be informed of the possibility of discovery of SF and the necessity to obtain written informed consent before genetic testing. The patient also always has the right not to know about SF results and can revoke their previously written consent about receiving such a result. They suggest that genetic laboratories are obliged to report SF from the minimum list to the clinician. The discovery of SF must be reported to the patient by a medical professional qualified in medical genetics.

**Diagram 1:**
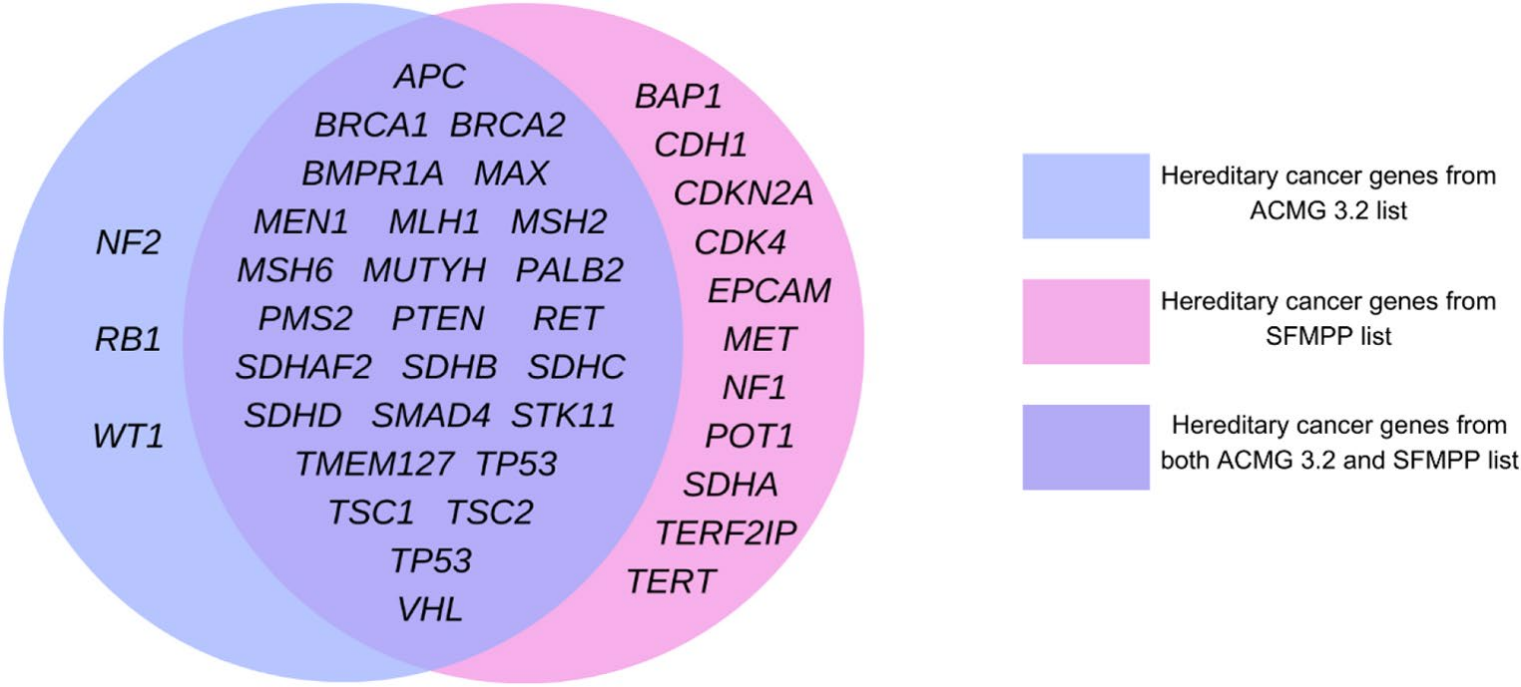
HC genes listed in the ACMG 3.2 list (blue), SFMPP list (pink) and both (violet). ACMG - American College of Medical Genetics and Genomics, SFMPP - French Society of Predictive and Personalized Medicine

The latest ACMG list and SFMPP minimum list for reporting SF differ slightly, HC genes from both lists are shown in Venn diagram below (Diagram 1).

## Aim of this systematic review

The growing use of genetic testing has resulted in a higher frequency of SF in HC genes. However, there is no clear guidance regarding the disclosure and clinical management of these findings, raising numerous ethical, clinical, and practical challenges. This systematic review aims to examine the frequency of SF reported in HC genes across diverse populations in different settings and real-world clinical practices regarding the reporting and management of SF in this context.

## Methods

This systematic review was prepared according to the Preferred Reporting Items for Systematic reviews and Meta-Analyses (PRISMA) statement (Page et al. 2021).

For the purpose of this systematic review, we use the term SF as we find it most suitable in this context, even though some previously published papers on this topic use other terms (incidental finding, additional finding, unsolicited finding).

### Search strategy

Comprehensive literature search was conducted on the electronic database PubMed from inception to June 2024 to evaluate current knowledge published about SF in human genetics after performing next generation sequencing. The search was limited to studies, published in English. We used the following search terms: secondary finding, incidental finding, additional finding, unsolicited finding, hereditary cancer, genetic test, genome/exome sequencing.

#### Inclusion criteria

Studies were included in this review if they had met the following inclusion criteria: (i) study design: single- or multicentre retrospective cohort studies, where a cohort of healthy or affected individuals underwent genetic testing for research or diagnostic purpose; (ii) intervention: genetic testing had to be performed with next-generation sequencing (WGS, WES or use of multi-gene panel test (MGPT)); (iii) outcome parameter: frequency of reported SF in HC genes had to be reported.

#### Exclusion criteria

We excluded studies where genetic testing was performed in the prenatal period or was focused on paediatric population only. We excluded recommendations, guidelines, case reports and reports of newly found genetic variations, studies of specific genetic diseases, studies involving genetic testing performed by another method, non-NGS based analyses (e.g., microarrays and karyotyping), studies of analysis of mitochondrial DNA, RNA analyses and pharmacogenetic SF.

Initially, a total of 2019 research papers were identified (search performed on July 15th, 2024). Titles of all found articles were screened for eligibility and 168 articles were selected for further selection based on abstract content. Full-text reading was made for 87 articles by a single researcher (EA) and according to inclusion and exclusion criteria 26 papers were selected for inclusion. Additional 4 research papers were identified by hand searching the reference lists of the included articles. A total number of included research papers was 30. The inclusion process is shown in Flowchart 1.

**Flowchart 1:**
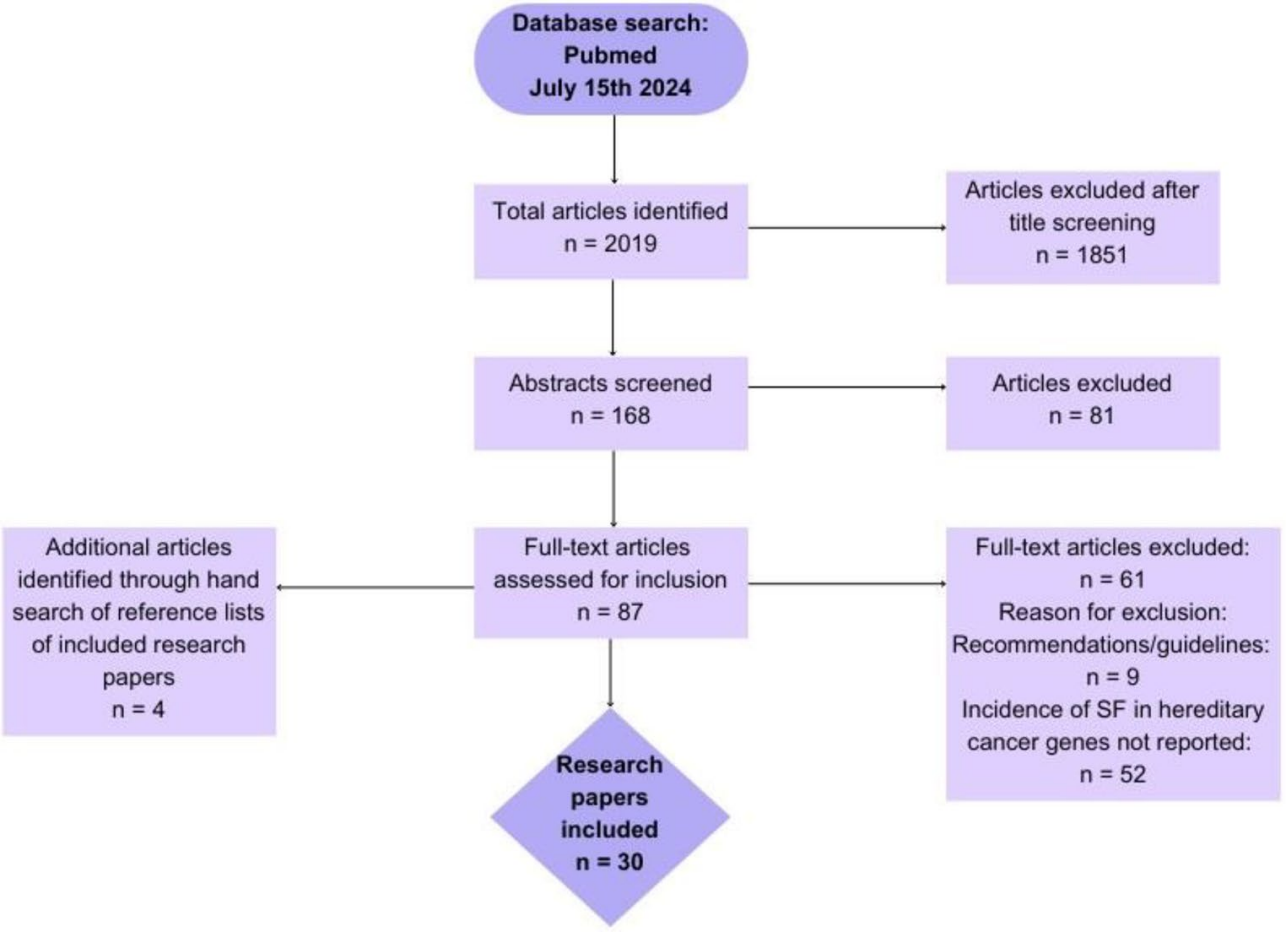
Flowchart of the systematic review according to the Preferred Reporting Items for Systematic Reviews (PRISMA) schema. PRISMA - Preferred Reporting Items for Systematic reviews and Meta-Analyses.

### Data extraction

We included 30 single- or multicentre retrospective cohort studies, which were published between 2012 and June 2024. From each of 30 included research papers the following data were extracted: author, publication year, sample size, purpose of genetic testing, and indication for genetic testing/cohort type or study in research setting, method of genetic testing, criteria for reporting SF, reported frequency of total SF and frequency of reported SF in HC genes. Additionally, the type of SF that were reported in HC genes and downstream information about their disclosure (psychological impact, cascade testing and surveillance related to SF in HC genes) were acquired where possible.

## Results

The extracted data is summarized in Table 1.

**Table 1:** Characteristics of included studies with reported frequencies of secondary findings

Author & Year of Publication	Country /Region	Sample size	Purpose of GT and indication for diagnostic GT / Cohort type or study in research setting		Method of GT	List of genes for reporting SF	Cases and frequency of all reported SF	Cases and frequency of reported SF in HC genes
Cheema et al. 2020	Pakistan	337	D	Suspected genetic disease	WES	ACMG 2.0	4 (1.2%)	1 (0.3%)
Arslan Ateş et al. 2021	Turkey	622	D	Suspected genetic disease	WES	ACMG 2.0	13 (2.1%)	7 (1.1%)
Carrasco et al. 2023	Spain	533	D	Suspected genetic disease	WES	ACMG 2.0 + additional HC genes	/	11 (2.1%)
Demir et al. 2024	Turkey	2020	D	Suspected genetic disease	WES	HC genes (ACMG 2.0 + others)	/	28 (1.4%)
Hart et al. 2019	Europe	6240	D	Suspected genetic disease	WES	ACMG 1.0	75 (1.2%)	25 (0.4%)
Jalkh et al. 2020	Lebanon	280	D	Suspected genetic disease	WES	ACMG 2.0	17 (6%)	3 (1.1%)
Jurgens et al. 2015	USA	232	D	Suspected genetic disease	WES	ACMG 1.0	2 (0.9%)	1 (0.4%)
Kuo et al. 2020	Taiwan	161	D	Suspected genetic disease	WES	ACMG 2.0	3 (1.9%)	2 (1.2%)
Martone. et al. 2022	Italy	383	D	Suspected genetic disease	WES	ACMG 2.0	21 (5.5%)	3 (0.8%)
Retterer et al. 2016	USA	2091	D	Suspected genetic disease	WES	ACMG 1.0	129 (6.2%)	19 (0.9%)
Yang et al. 2014	USA	2000	D	Suspected genetic disease	WES	ACMG 1.0 + other medically actionable genes	95 (4.8%)	25 (1.3%)
Henn et al. 2019	Germany	237	D	Suspected or known hereditary tumour syndrome	MGPT (148 HC genes)	All HC genes from selected MGPT	/	5 (2%)
Nambot et al. 2021	France	2500	D	Suspected hereditary tumour syndrome	MGPT (47 HC genes)	All HC genes from selected MGPT	/	14 (0.6%)
Jensson et al. 2023	Iceland	57,933	R	Disease projects at deCODE Genetics	WGS	ACMG 3.0	2348 (4.1%)	982 (1.7%)
Tang et al. 2018	East Asia	954	R	Patients with Hirschprung disease and healthy controls	WGS	ACMG 2.0	24 (2.5%)	8 (0.8%)
Thompson et al. 2018	USA	789	R	Healthy individuals	WGS	ACMG 2.0 + other medically actionable genes	25 (3.2%)	8 (1.0%)
Horiuchi et al. 2021	Japan	2480	R	Project HOPE – patients after surgical treatment of various cancer types	WES	ACMG 2.0 + 13 additional HC genes	36 (1.5%)	25 (1.0%)
Amendola et al. 2015	Europe & Africa	6503	R	NHLBI Exome Sequencing Project	WES	Medically actionable genes	113 (1.7%)	27 (0.4%)
Yu et al. 2021	Hong Kong	1116	R	Healthy individuals	WES	ACMG 2.0	17 (1.5%)	7 (0.6%)
Haer-Wigman et al. 2019	Netherlands	1640	R	Healthy individuals	WES	ACMG 2.0	44 (2.7%)	11 (0.7%)
Jang et al. 2015	Korea	196	R	Patients with various non-genetic diseases and healthy controls	WES	ACMG 1.0	13 (6.6%)	6 (3.1%)
Kasak et al. 2022	Europe & North America	836	R	GEMINI NOA cohort	WES	ACMG 2.0 + other medically actionable genes	30 (3.6%)	10 (1.2%)
Kwak et al. 2017	Korea	1303	R	KOEX study cohort	WES	ACMG 1.0	32 (2.5%)	9 (0.7%)
Lawrence et al. 2014	USA	159	R	NIH Undiagnosed Diseases Program	WES	ACMG 1.0	14 (8.8%)	4 (2.5%)
Van Rooij et al. 2020	Netherlands	2628	R	Healthy elderly population	WES	ACMG 2.0	26 (1.0%)	7 (0.3%)
Nolan et al. 2024a	UK	17,194	R	UK 100KGP	MGPT (HC + FH genes)	All genes from selected MGPT	157 (0.9%)	83 (0.5%)
Olfson et al. 2015	Europe, Africa, East Asia, Americas	1092	R	1000 Genomes	MGPT (ACMG 1.0)	ACMG 1.0	12 (1.1%)	4 (0.4%)
Johnston et al. 2012	USA	572	R	ClinSeq participants with atherosclerosis phenotype	MGPT (37 HC genes)	All HC genes from selected MGPT	/	4 (0.7%)
Haverfield et al. 2021	USA	10,478	R	Participants in proactive genetic screening	MGPT (147 genes)	All genes from selected MGPT	649 (6.2%)	329 (3.1%)
Gordon et al. 2020	USA	21,915	R	eMERGE III	MGPT (109 genes)	All genes from selected MGPT	661 (3.0%)	261 (1.2%)

GT – genetic testing, D – diagnostic, R – research, WES – whole exome sequencing, WGS – whole genome sequencing, ACMG - American College of Medical Genetics and Genomics, HC – hereditary cancer, MGPT – multi-gene panel test, FH – familial hypercholesterolemia

Inclusion criteria were different among the included research papers. Some papers collected data from genetic analyses of patients, who required genetic testing for a suspected genetic condition. In other studies individuals were genetically tested as a part of larger genomic population studies for research purposes. Three of the included studies (Henn et al. 2019; Horiuchi et al. 2021; Nambot et al. 2021) report the frequency of SF in HC genes after germline genetic testing in cancer patients and in patients with known or suspected hereditary tumour syndrome

There was a wide span of reported SF overall as well as in HC genes in the included studies ranging from 0.9–8.8% and 0.4–3.1%, respectively

Altogether the studies included 145,424 participants who underwent genetic testing and a total of 1929 SF in HC genes were reported. This accounts for 1.3% of all included participants, in other words a SF was discovered 1 in 75 participants

## Informed consent for reporting SF

Nine of the included studies reported the rate of individuals who gave informed consent for reporting SF before they underwent genetic testing. The rates are reported in Table 2 and vary from 69 to 100%.

**Table 2:** Rate of individuals that gave informed consent for reporting

	Cheema et al. 2020	Carrasco et al. 2023	Horiuchi et al. 2021	Jalkh et al. 2020	Retterer et al. 2016	Nambot et al. 2021	Thompson et al. 2018	Amendola et al. 2015	Nolan et al. 2024a
Rate of individuals who opt in for disclosure of SF (%)	96.5	92	69	100	88	100	85	98	92

SF – secondary findings

## Type of secondary findings in hereditary cancer genes

**Graph 1:**
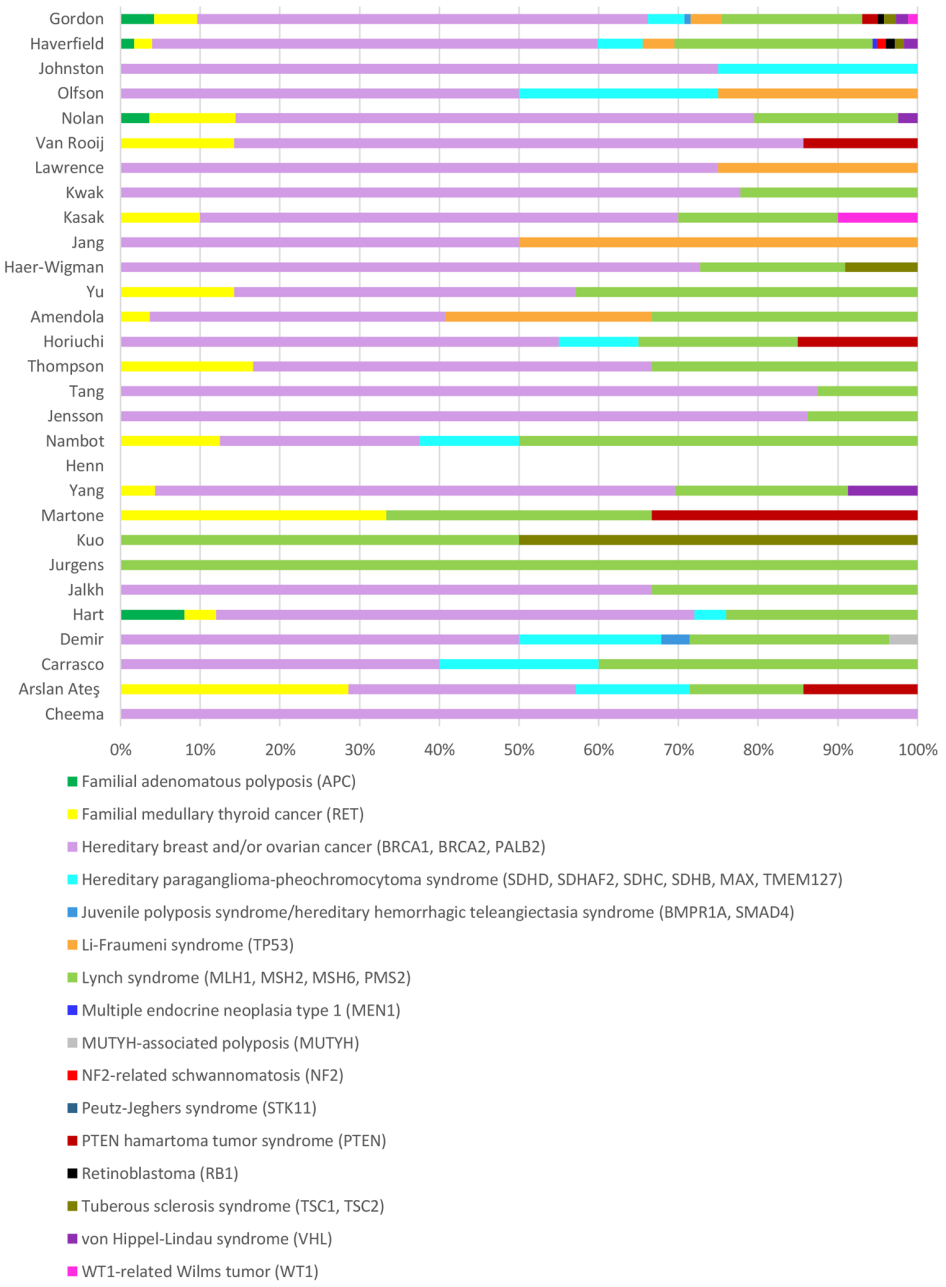
Frequency of reported SF in HC genes from ACMG 3.2 list in all the included studies. SF – secondary findings, HC – hereditary cancer, ACMG - American College of Medical Genetics and Genomics

Apart from the study from Retterer et al. (2016), all the included studies reported which HC genes were reported as SF, which is shown in Graph 1. The most commonly reported SF are from the hereditary breast and/or ovarian cancer (HBOC) gene list (72%), followed by Lynch syndrome genes. Eight studies also reported SF in HC genes that are not part of the ACMG 3.2 list. This is not shown in Graph 1 for better visual effect of prevalence of different types of SF. The majority of these additional SF were found in *NF1*,* CDH1*,* CDKN2A*,* RAD51C*,* RAD51D*,* SDHA* and *EPCAM*. Most of them are potentially medically actionable and included in the minimum SFMPP list for reporting SF.

The number of SF in HC genes were only reported for 855/982 of total SF in HC genes in the study from Jensson et al. (2023), therefore there is only data for the 855 of the reported SF in the Graph 1 and the study from Henn et al. (2019) reported only SF which are not on the list.

## Disclosure of Sf in cancer genes and patient response in practice

**Table 3:** Summary of additional information about disclosure of SF in HC genes

Author & Number of patients with available data/total patients with reported SF	Personal history of cancer	Family history of cancer	Psychological impact	Cascade testing of relatives & Diagnostic yield	Surveillance	Result of surveillance
Carrasco et al. 2023 11/11	/	/	32 index cases & tested relatives participated – statistically significant higher levels of uncertainty and distress were observed in comparison with controls	42 relatives tested: 1st degree:23 2nd degree: 9 3rd degree: 10 18/42 were positive	20/29 enrolled in surveillance program, 9 were excluded due to their age	4/20 diagnosed with cancer related to their SF 3/20 underwent prophylactic risk reduction surgery 1/20 was a candidate for targeted therapy for metastatic cancer 1/20 had polyps removed in colonoscopy
Demir et al. 2024 9/28	/	3 index cases had close relatives with cancer related to their SF	/	18 relatives tested	All positive for SF enrolled in surveillance program	3 diagnosed with cancer related to their SF 1 had polyps removed in colonoscopy
Hart et al. 2019 9/25	34% of all participants with SF (not just HC genes) had positive family history for the reported SF		No one voiced regret about finding out about SF	All 9 reported their SF to their families; relatives of 3/9 opted for GT	Undercompliance with recommended surveillance programme	/
Horiuchi et al. 2021 20/25	11/20 (55%)	10/20 (50%)	/	Relatives of 4 patients with SF in HC genes opted for GT; some relatives were positive	15/20 patients chose medical consultation and/or follow up surveillance	1 relative was diagnosed with early-stage cancer
	7/20 (35%) had personal and family history of cancer					
Thompson et al. 2018 8/8	4/8 (50%) had family or personal history of cancer; all 4 would have met the criteria for genetic testing via clinical guidelines		/	/	/	/
Nolan et al. 2024a 57/83	3/57 (5%)	14/57 (25%)	SF was unexpected for most, many did not remember discussing or giving consent, expressed anxiety and worry for relatives, some expressed gratitude; clinical surveillance offers reassurance; most did not regret their decision to receive SF (Nolan et al. 2024b)	/	All patients with disclosed SF were referred for surveillance, some attended recommended surveillance program	Results of follow up after 4 years: 1/57 patient diagnosed with cancer related to SF after follow up 11/25 patients opted for risk reducing surgeries 3/3 patients had polyps found at surveillance colonoscopy
	20/57 (35%) had personal and/or family history					

SF – secondary findings, GT – genetic testing, HC – hereditary cancer

Six of the included studies reported additional information about disclosure of SF in HC genes in 114 patients. The summary is shown in Table 3.

Personal and/or family history of cancer was present in 5–55% of patients with discovered SF. Nolan et al. (2024a) and Carrasco et al. (2023) report higher levels of stress after disclosure of SF, but patients mostly did not regret finding out about SF (Hart et al. 2019; Nolan et al. 2024b). Nolan et al. (2024a) additionally described the clinical surveillance to be reassuring for patients with discovered SF. Four studies report that at least 67 relatives of 49 patients with reported SF decided for cascade genetic testing. Compliance with surveillance programs varies between included studies from 0 to 100%, but it is clear that an important number of patients with disclosed SF do attend surveillance and some undergo prophylactic surgeries. There were at least 9 diagnoses of cancer related to the SF in a short observation period.

## Discussion

The results of the studies included in this systematic review indicate that the majority of patients who undergo genetic testing opt in for disclosure of medically actionable SF. It is important to keep in mind that a minority of individuals does not agree with reporting of such findings and it is therefore necessary to give them a chance to opt out for disclosure of these findings during pre-test genetic counselling.

As is true for all SF, the frequency of discovered SF in HC genes is not negligible. Our assessment of reported SF predicts that an important, potentially medically actionable genetic finding in HC genes was unexpectedly found in approximately 1 in 75 participants from all the included studies, which correlates with expected prevalence of 2% HC syndromes in presumably healthy subjects (Imyanitov et al. 2023). Most commonly reported SF are from the HBOC syndrome gene list, followed by the SF in Lynch syndrome genes. This is consistent with the fact that these two syndromes are the most prevalent HC syndromes.

As HBOC syndrome and Lynch syndrome contribute the most to HC morbidity and mortality, such findings could be important for planning individualised preventive measures and consequently reduce the lifetime morbidity and mortality from cancer similar to other cancer screening programs. This is a strong argument in the widely debated topic of implementing opportunistic screening.

There is limited data about the impact of disclosure of SF in HC genes. The existing knowledge about this topic suggests that although the revelation of SF can cause anxiety and stress, none of the participants voiced regret about finding out about SF. Less than half of all patients who received such a finding reported a personal and/or family history of cancer, related to their SF. The majority of these patients would therefore not be eligible for genetic testing for this particular HC syndrome. The participation in surveillance programs for SF might not be as high as for primary findings, but from the limited data from the studies included in this review we can see that a substantial number of patients follows the surveillance programs and some of them receive a diagnosis of early-stage cancer related to their SF after undergoing recommended surveillance/risk reducing surgeries. Even though the surveillance period for patients in these studies was short, these diagnoses indicate there is a tangible health benefit from disclosing SF to carriers.

The aim of our manuscript was to review studies that reported SF in HC genes and to summarize the data presented in those studies. However, definitions and reporting practices for SF may vary across authors, and as a result, some findings described as SF in the included studies might not align with the definition of SF we outlined in our Introduction. A subset of the included studies (Henn et al. 2019; Horiuchi et al. 2021; Nambot et al. 2021) reported SF in HC genes among cancer patients and individuals with known or suspected hereditary tumour syndromes. We consider this a limitation in interpreting the data from these studies, as there is currently no definitive scientific evidence proving that the pathogenic or likely pathogenic variants identified as SF in such contexts are unrelated to the patients’ cancer diagnoses. Furthermore, uncertainties remain regarding the full spectrum of cancer associated with hereditary tumour syndromes and the penetrance of pathogenic or likely pathogenic variants in HC genes identified to date.

Acquired knowledge so far suggests that opportunistic screening with the use of NGS for HC syndromes could serve as a beneficial tool for finding individuals at high risk for developing cancer. Limited information from the included studies show none to minimal negative impact on patients receiving SF in HC genes and it has also been proven before that such screening for HC syndromes is cost-effective (Dioun et al. 2024). Nevertheless, many limitations from patient and provider perspectives still remain before implementing genomic screening to everyday practice and this is still broadly discussed among geneticists worldwide (Pederson and Narod 2024).

## Conclusion

The results of our review suggest that reporting the SF from the ACMG minimum list enables health care providers to find a substantial number of carriers of highly penetrant pathogenic variants in HC genes.

The disclosure of SF after germline genetic testing still presents a complex interplay of clinical, ethical, and practical challenges. While guidelines and recommendations provide a framework, further research of real-life experiences of providers and patients are needed for optimizing the integration of SF disclosure and its consequent patient surveillance into clinical care in existing healthcare systems.

## Data Availability

No datasets were generated or analysed during the current study.
